# Contrasting water-use patterns of Chinese fir among different plantation types in a subtropical region of China

**DOI:** 10.3389/fpls.2022.946508

**Published:** 2022-09-15

**Authors:** Ying Zhang, Qing Xu, Beibei Zhang, Deqiang Gao, Ting Wang, Wenbin Xu, Ranran Ren, Silong Wang

**Affiliations:** ^1^Key Laboratory of Forest Ecology and Environment of National Forestry and Grassland Administration, Ecology and Nature Conservation Institute, Chinese Academy of Forestry, Beijing, China; ^2^Huitong Experimental Station of Forest Ecology, Institute of Applied Ecology, Chinese Academy of Sciences, Shenyang, China

**Keywords:** stable isotope, plantation types, water-use patterns, Chinese fir, plant and soil properties

## Abstract

Plantation cultivation plays an important role in improving terrestrial ecosystem functions and services. Understanding the water-use patterns of major afforestation species is vital for formulating ecological restoration strategies and predicting the response of plantation to climate change. However, the impacts and drivers of forest types on water-use patterns of key tree species are poorly understood. Here, the combined methods of dual stable isotope of *δ*D and *δ*^18^O and Bayesian mixed framework (MixSIAR) were employed to investigate the water-use patterns of *Cunninghamia lanceolata* (Chinese fir) in a monoculture, mixed forest with *Cinnamomum camphora*, and mixed forest with *Alnus cremastogyne* under different rainfall events in subtropical China. Furthermore, the relative contribution of different soil and plant factors to the water-use patterns of Chinese fir was quantified using a random forest model. Our results showed that Chinese fir in the mixed forests (with *C. camphora* or with *A. cremastogyne*) utilized less water from shallow soil compared to that in a monoculture but significantly improved the proportion of water absorbed from deep soil with the increase of 55.57%–64.90% and 68.99%–108.83% following moderate and heavy rainfall events, respectively. The most important factors contributing to the differences in water-use patterns of Chinese fir among monoculture and mixed forests were tree attributes (i.e., leaf biomass, eco-physiological regulation, and fine root biomass). These findings reveal that Chinese fir in mixed forests could optimize water-use patterns by adjusting plant properties for interspecific niche complementarity, improving the utilization of deep soil water. Overall, this study suggests that mixed-species plantations could improve water-use efficiency and reduce the sensitivity of tree species to precipitation change, indicating they are better able to cope with expected climate variability.

## Introduction

Plantations exert a crucial role in terrestrial ecosystem function by providing multitudes of products and services (Law et al., 2018). However, planted forests in many areas are expected to experience an increase in the frequency and intensity of seasonal drought and extreme precipitation episodes (Schlaepfer et al., 2017; Taylor et al., 2017), which may considerably affect the structural function and sustainability of forest ecosystems (Hammond et al., 2022). Water-use patterns are critical for sustaining vegetation growth and productivity, particularly under the context of global change (Breckle, 2002; Barbeta et al., 2015). Generally, the flexibility of plants to use deep soil water may help them adapt to variable environments, as deep roots could contribute more water for plant transpiration than shallow roots during droughts (Bleby et al., 2010), and are more conductive to the stability of plant communities. By comparison, tree species that depend solely on shallow soil water could suffer from irreversible mortality due to extreme precipitation and drought episodes (Mcdowell and Allen, 2015; Ehbrecht et al., 2021). Chinese fir (*Cunninghamią lanceolata*) is one of the foremost afforestation tree species in the subtropics of China (Wang et al., 2008), reaching 9.90 × 10^6^ hm^2^ in scale and ranking first among all afforestation species (SFA, 2019). Furthermore, this tree species plays an essential role in increasing timber production because of its rapid growth and excellent timber quality (Wang et al., 2008). Thus, its growth characteristics and ecological functions have aroused considerable interest among ecologists (Liao et al., 2019; Farooq et al., 2020). However, to date, the patterns and mechanisms of water use strategies in Chinese fir remain unclear. Filling this knowledge gap will not only help to elucidate plantation-ecosystem processes, structures, and functions, but also provide guidance for the management of planted forests in response to climate change, which is crucial given the increasing expansion of subtropical plantations.

Compared to pure forests, plantations with mixed tree species seem to be a more effective management strategy to mitigate the impacts of climate change (Kelty, 2006; Lebourgeois et al., 2013), as the dominant tree species in mixed forests are more productive and less sensitive to climate change than in monocultures (Río et al., 2017; Muñoz-Gálvez et al., 2021). In terms of water-use characteristics, it has been reported that mixed species can reduce water competition and promote stable coexistence through hydrological niche differentiation or functional complementarity (Silvertown et al., 2015). For example, Wang et al. (2020) reported that owing to water source segregation, mixed forests decreased the competition of *Armeniaca sibirica*, *Ailanthus altissima*, and *Robinia pseudoacacia* for soil water in semiarid ecosystems. Similarly, Bello et al. (2019) showed that *Pinus sylvestris* in mixed temperate forests reduced water constraints for *Quercus petraea* by shifting water sources to deeper soils. Moreover, some studies also demonstrated other facilitation processes, such as hydraulic lift by deep-rooted species supplying water to neighboring plants (Emerman and Dawson, 1996; Brooks et al., 2006; Rodríguez-Robles et al., 2015). Nevertheless, if mixed and co-existing species share similar functional characteristics and adopt similar water-use strategies, ecological niche overlap occurs (Yang et al., 2015; Magh et al., 2020), which could lead to water competition and ultimately increase the degree of drought. Grossiord et al. (2014a) found that *Quercus petraea* increased the intensity of water stress of *Quercus cerris* in mixed Mediterranean forests. Hence, the effects of plantation types on plant water-use patterns are still being debated, which may be related to differences in the local climate, environment, and tree species. In addition, inconsistent results have been found in studies concerning water uptake patterns of the same tree species in monocultures and mixtures. For instance, Schume et al. (2004) found that European beech (*Fagus sylvatica*) occupied a large proportion of the deep-rooting zone in a mixture with Norway spruce (*Picea abies*), thus increasing the utilization of deep soil water. However, a recent study has demonstrated that neither species diversity nor competition intensity clearly affect the water uptake depth for European beech in mixed forests (mean absorption depth of 20–30 cm soil layer; Grossiord et al., 2014b). Overall, whether and how Chinese fir adjusts water-use patterns to cope with the shift from pure to mixed forests is still unclear, thereby limiting the understanding of water management in Chinese fir plantations.

Plant water-use patterns are commonly associated with multiple factors (Coners and Leuschner, 2005). Typically, plant and soil properties are the principal factors controlling water-use patterns (Zhou et al., 2019). It has been reported that the biomass and morphological structure of functional root systems play a critical role in influencing plant water uptake (Fort et al., 2017; Regine et al., 2018). For instance, dimorphic root systems have been observed in many tree species, permitting plants to absorb water from different soil depths through shallow, lateral, and deep roots (Dawson and Pate, 1996). Furthermore, physiological studies suggest that stomatal regulation (i.e., stomatal conductance) and leaf water potential (reflecting tree physiological activity and water status) are important factors influencing plant water-use strategies (West et al., 2007; Stahl et al., 2013). Leaf biomass may also affect plant water-use patterns via regulating transpiration (Ouyang et al., 2020). Aside from plant properties, other research has highlighted the key role of soil properties in controlling plant water-use patterns (Sperry and Hacke, 2002; Meißner et al., 2014). Soil hydraulic parameters (i.e., field capacity, total porosity) can shape soil water storage and spatiotemporal distribution (Xu and Li, 2008; Yang and Fu, 2017), thereby affecting the water-use patterns of overstory trees. In general, though many studies have explored the factors influencing plant water-use patterns, the relative contributions of these biotic and abiotic drivers to plant water-use patterns are poorly understood. Given the variations in the soil water holding capacity between monoculture and mixed-species plantations (Zhang et al., 2021), we speculated that soil properties were the main drivers influencing water-use patterns of Chinese fir in different plantation types. To test this hypothesis, the impacts of the above-mentioned soil and plant properties on the water-use patterns of Chinese fir are urgently needed.

The stable isotope technique is a non-destructive and powerful tool used to identify potential water sources utilized by plants, as hydrogen (*δ*D) and oxygen (*δ*^18^O) isotopic fractionation is absent during root water uptake and conduction of water from roots to leaves in terrestrial plants (Dawson and Ehleringer, 1991), with the exception of a few halophytic plants (Lin and Sternberg, 1993) and woody xerophyte plants (Ellsworth and Williams, 2007). Hence, this technology has been widely applied to investigate the water-use characteristics of plants in different terrestrial ecosystems (Cheng et al., 2006; Geißler et al., 2019; Liu et al., 2019; Sohel et al., 2021). In this study, stable deuterium (*δ*D) and oxygen (*δ*^18^O) isotope techniques were employed to analyze water-use strategies of Chinese fir under different plantation types. We aimed to address the following two questions: (1) Do different plantation types (pure and mixed plantations) affect the water-use patterns of Chinese fir? and (2) What factors determine water-use patterns of Chinese fir?

## Materials and methods

### Study site

This work was carried out at the Huitong National Research Station of Forest Ecosystem of the Chinese Academy of Sciences (26°51′N, 109°36′E, elevation 300–1,100 m), which is based in a subtropical monsoon zone. The mean annual precipitation varies between 1,200 and 1,400 mm, with ~67% occurring in the growing season from April to August (Wang et al., 2008). Relative humidity and mean annual temperature are 80% and 16.5°C, respectively. The soil of this region is categorized as Typic Dystrudept under the U.S. Soil Taxonomy (the second edition), and detailed information on the soil characteristics is described in Supplementary Table S1. The original zonal vegetation was a subtropical evergreen broad-leaved forest, which was almost destroyed due to the influence of anthropogenic activities, and Chinese fir plantation has dominated the forest community in the region (Wang et al., 2008).

Three different Chinese fir plantations were set up in the early spring of 1990 at an altitude of about 500 m a.s.l. after a clear-cut of first-generation Chinese fir forest in 1989, including (i) pure Chinese fir forest (PC); (ii) a mixed forest of Chinese fir with *Cinnamomum camphora* (MCC); and (iii) a mixed forest of Chinese fir with *Alnus cremastogyne* (MCA). The mixed ratio of *C. lanceolata* to broad-leaved tree species was 8:2 in both mixtures. In addition, the planting density of all plantations was 2000 stems ha^−1^, and the total planting area was about 7.5 ha. The slope aspect of these plantations was southwest, with slope grades of 24°–26°. All plantations were established with similar soil properties (Wang et al., 2008). In our study, three replicate plots (20 m × 20 m, 400 m^2^) with the homogeneous visual structure and composition were randomly built in each Chinese fir stand. All of the plots established in each Chinese fir plantation were separated from each other by >20 m. There was no human interference during the entire experiment apart from investigation and sampling.

### Field sampling

According to the meteorological classification criteria of light (precipitation 0–10 mm per 24 h), moderate (precipitation 10–25 mm per 24 h), and heavy rainfall (precipitation >25 mm per 24 h), the following three representative rainfall events were selected during the sampling period: (i) 8.5 mm on the 1st August 2020; (ii) 15.5 mm on 18th September 2019; and (iii) 36.9 mm 26th September 2019. In the present study, rainwater, soil, and xylem samples were collected. There was no occurrence of precipitation during the sampling period. To collect rainfall samples, a rain gauge cylinder was randomly placed in an open space near each plantation site. After each rainfall event, rainwater from these three cylinders constituted a mixed homogeneous sample and was immediately placed into 4 ml glass vials. Xylem and soil samples were collected every other day after each rainfall event and before the next rainfall event. To collect xylem samples, we selected three healthy Chinese fir with similar crown sizes and diameters at breast height (DBH, 1.3 m) from each plot. Three mature and healthy xylem samples, 3–4 cm in length, were collected from each selected tree. Meanwhile, soil samples were obtained near the sampled trees by a soil corer (diameter 5 cm) at five layers (0–20, 20–40, 40–60, 60–80, and 80–100 cm). A portion of soil samples gathered from each layer was put into glass vials for hydrogen and oxygen isotope measurements; the remaining portion was stored in an aluminum box used for the soil water content (SWC, %) analysis. All samples for isotope analysis were immediately put into the glass vials (8 ml glass vials for xylem samples, 4 ml glass vials for rainwater and soil samples), quickly sealed with parafilm, and put in a transportable cooler. After sampling, all glass vials with samples were kept frozen in the refrigerator (−18°C) before water extraction and isotopic determination.

### Measurement of plant and soil parameters

Predawn leaf water potential (ψ_pd_) and gas exchange parameters of Chinese fir in the three plantations were measured simultaneously during the sampling period. The specific meteorological variables of the study site on the measurement dates are presented in Supplementary Table S3. After each rainfall event, three healthy Chinese firs with similar crown size and DBH were randomly selected to investigate ψ_pd_ and other physiological parameters in each plot. As for the ψ_pd_, it was determined by a WP4C Dewpoint Potential Meter (Decagon, United States) before sunrise from 5:00 a.m. to 6:00 a.m. To minimize water loss, ψ_pd_ was immediately measured in a WP4C sample chamber after collecting leaves from the middle of the canopy. The measurement precision of the WP4C was ±0.05 MPa. Leaf gas exchange parameters were determined under natural conditions using a portable gas-exchange system (IRGA; Li6400; LI–COR Inc., Lincoln, United States) on cloudless days from 9:00 a.m. to 11:00 a.m. When ΔCO_2_ fluctuated <0.2 μmol·mol^−1^, the photosynthetic parameters varied <0.1 μmol·mol^−1^, indicating that the leaves attained a physiological steady-state, and only then were the values recorded. Five branches and five mature leaves for each selected individual were measured. To accurately determine the photosynthetic area of conifers, the area of the leaf chamber covered by the needles was scanned with an Epson Perfection V700 Photo Scanner after the above measurements. The leaf gas exchange parameters (photosynthetic rate: P_n_; stomatal conductance: G_s_; and transpiration rate: T_r_) were recalculated based on the actual leaf area. The leaf biomass (LB) of Chinese fir was determined using an allometric growth model based on DBH. In addition, fine root samples (<2 mm in diameter) in five layers were gathered from 30 cm × 30 cm × 20 cm quadrats in three directions (with 120° intervals), at ~50–100 cm from base of the trunk. After being washed free of soil, all fine root biomass samples were dried at 75°C in an oven and weighed.

We also assessed soil properties of the Chinese fir plantations on 23 July 2019 (Supplementary Table S3). Firstly, soil samples were obtained from five layers (0–20, 20–40, 40–60, 60–80, and 80–100 cm) with stainless steel containers (volume in 100 cm^3^). Soil physical parameters (i.e., field capacity, total porosity) were then determined via the use of a cutting ring method (Özcan et al., 2013). The soil fertility characteristics of the three Chinese fir plantations are shown in Supplementary Table S2.

### Sample pretreatment and the isotopic composition determination

The water in the plant xylem and soil samples was obtained via a cryo-vacuum distillation extraction system (Ehleringer et al., 2000). The isotopic compositions (^18^O/^16^O or D/H) of captured xylem water, soil water, and rainwater were determined using an isotope ratio mass spectrometer (Delta V Advantage) connected to an elemental analyzer (Flash 2000 HT; Thermo Fisher Scientific, Inc., Waltham, United States). The measurements for *δ*D and *δ*^18^O had a precision of ±1‰ and ±0.2‰, respectively. The isotopic ratios of *δ*D and *δ*^18^O are denoted in standard delta notion (*δ*) as the following formula:


$$\delta =\left(\frac{R_{\mathrm{sample}}}{R_{\mathrm{standard}}}-1\right)\times 1000‰$$

where *R*_sample_ is the molar ratios of heavy (^18^O or D) to light (^16^O or H) isotope in the measured water samples, and *R*_standard_ represents the Vienna Standard Mean Ocean Water standard.

### Calculation of the percentage of water uptake in Chinese fir

The Bayesian isotope mixing framework (MixSIAR) was introduced to estimate the percentage of water used by Chinese fir from potential water sources. Specifically, the isotope values from the xylem water of Chinese fir and soil water of each layer were imported to the model as the mixture and source data, respectively. The discrimination factors for *δ*D and *δ*^18^O were set to zero, and the run length of the “MCMC (Markov chain Monte Carlo)” was set to “long.” Furthermore, “uninformation/Generalist” and “Residual only” were designed as the specified priority and error structure. The convergence of the model was tested by “Gelman-Rubin” and “Geweke” (Wang et al., 2017).

### Statistical analysis

One-way analysis of variance (ANOVA) was performed general linear model with the Duncan as *post hoc* multiple comparison to test the differences in hydrogen (*δ*D) and oxygen (*δ*^18^O) isotopic compositions of xylem water, as along with the percentage of water uptake by Chinese fir among different plantation types. The significance level of the statistical tests was set at 0.05. Subsequently, correlation analysis was employed to investigate relationships between the plant, soil properties, and percentage of water uptake by Chinese fir. To discern the dominant factors affecting the percentage of water absorbed by Chinese fir, we then employed random forest analysis using the “randomForest” package in R software (version 4.1.0). In these random forest analyses, percentage increases in the mean squared error (MSE) for each factor were used to represent the relative importance of each predictor. In addition, the significance of each predictor was assessed with the “rfPermute” package (Archer, 2016). The statistical analysis was carried out with R 4.1.0.

## Results

### Precipitation, xylem water, and soil water isotopic composition

**Figure 1:**
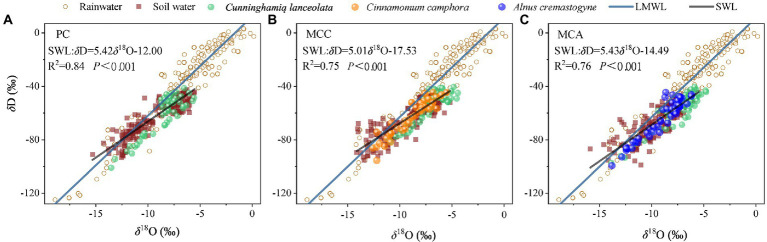
Scatter plots of hydrogen (*δ*D) and oxygen (*δ*^18^O) in soil water and xylem water in different plantation types: **(A)** PC, pure Chinese fir stand; **(B)** MCC, mixed stand with *C. camphora*; and **(C)** MCA, mixed stand with *A. cremastogyne*.

**Figure 2:**
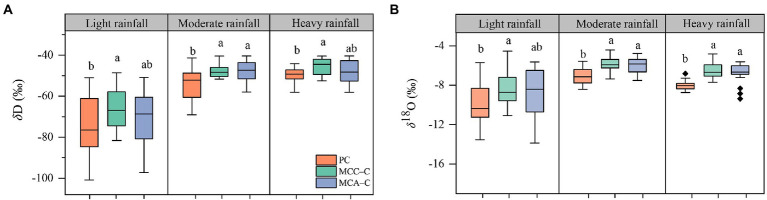
The differences of **(A)** *δ*D and **(B)** *δ*^18^O xylem water for Chinese fir in monoculture (PC), mixed forest with *C. camphora* (MCC-C), and mixed forest with *A. cremastogyne* (MCA).

The local meteoric water line (LMWL; *δ*D = 7.43*δ*^18^O + 12.39; *R*^2^ = 0.90, *p* < 0.01) was fitted according to the rainwater isotope data during the sampling period (October 2017–October 2020). As shown in Figure 1, the LMWL had a lower slope and similar intercept value than the global meteoric water line (GMWL; *δ*D = 8*δ*^18^O + 10), suggesting that rainwater underwent different degrees of evaporation enrichment during rainfall. In addition, most of the data points of *δ*D and *δ*^18^O of the soil water for these forest plots were found on the right side of the LMWL. The *δ*D and *δ*^18^O for xylem water in the Chinese fir plotted close to that area in the soil water, indicating that the Chinese fir predominantly derived water from the soil. Moreover, there were significant differences in the isotopic compositions of hydrogen (*δ*D) and oxygen (*δ*^18^O) for the Chinese fir xylem water for the three forests after each rainfall event (Figure 2). Specifically, *δ*D values of Chinese fir xylem water in PC were lower than those in MCC and MCA, although a significant difference was only found following the moderate (15.5 mm) rainfall event (Supplementary Table S4). However, the *δ*^18^O values of Chinese fir xylem water in mixed forests (MCC and MCA) were significantly greater than those in the pure forest (*p* < 0.05; Supplementary Table S4) following moderate and heavy rainfall. These results suggested that Chinese fir in different plantation types took up water from different soil layers.

### Water absorption percentage of Chinese fir

**Figure 3:**
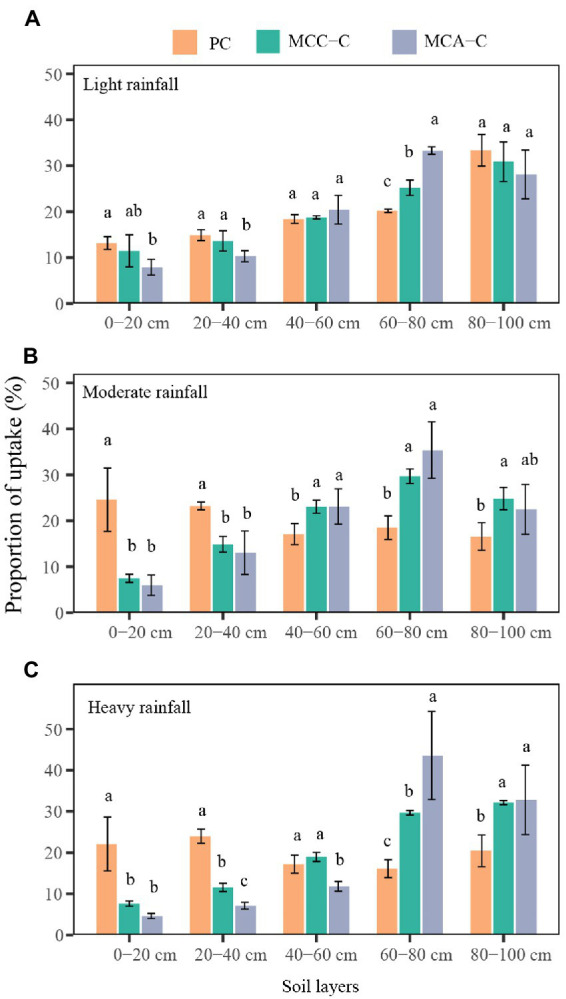
Variations in percentage of water uptake by Chinese fir in different plantation types after **(A)** light, **(B)** moderate and **(C)** heavy rainfall. Error bars denote one standard deviation. Lowercase letters indicate significant differences of the percentage of water uptake by Chinese fir in different plantation types (*p* < 0.05). PC, Chinese fir in the pure forest; MCC–C, Chinese fir in the mixed forest with *C. camphora*; MCA–C, Chinese fir in the mixed stand with *A. cremastogyne*.

The percentage of water absorbed by Chinese fir from different soil layers in the three plantations was determined using the MixSIAR model, based on both *δ*D and *δ*^18^O values (Figure 3). Following light rainfall, no significant difference was detected among the three plantations with respect to the water that Chinese fir acquired within the 0–60 cm and 80–100 cm soil layers. However, Chinese fir in the mixed forests (MCC and MCA) derived a higher percentage of water within the 60–80 cm soil layer than that in the pure forest (*p* < 0.05; Figure 3). Moreover, Chinese fir in MCC and MCA primarily acquired water in deep soil layers (60–100 cm), with the utilization percentages of 54.56% and 61.80% during moderate rainfall and 57.83% and 76.37% during heavy rainfall. Conversely, the percentage of the shallow soil water (0–40 cm) extracted by Chinese fir in the pure forest reached 47.83% and 46.13% after moderate and heavy rainfall, respectively, which was significantly higher than that in the mixed forests.

### Characteristics of plant and soil for the three Chinese fir plantations

**Figure 4:**
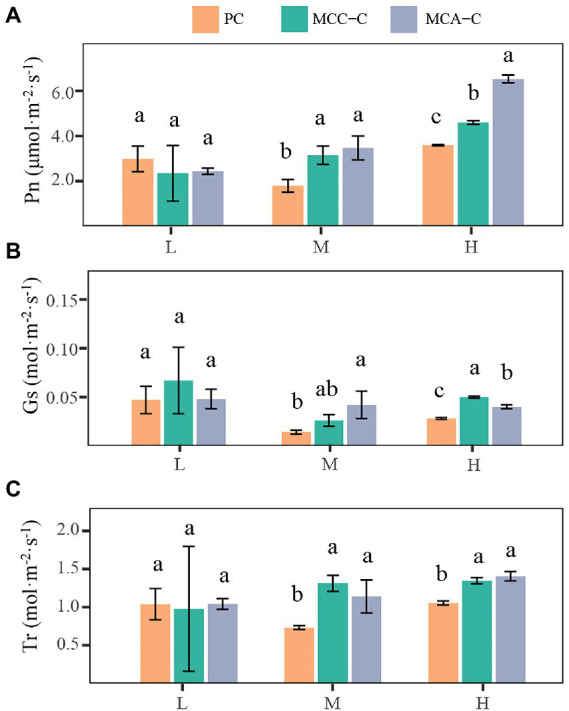
Leaf gas change parameters, including Pn **(A)**, Gs **(B)**, and Tr **(C)**, for Chinese fir in pure (PC) and mixed plantations (MCC and MCA) after three rainfall events. Error bars denote the standard deviation. Lowercase letters indicate significant differences of gas exchange parameters for Chinese fir among the three plantations (*p* < 0.05). L, light rainfall; M, moderate rainfall; L, light rainfall; P_n_, photosynthetic rate; G_s_, stomatal conductance; T_r_, transpiration rate.

The properties of Chinese fir differed by plantation type. Among the six biotic factors, the LB of Chinese fir in the mixed forests (MCC and MCA, respectively) was significantly greater than that in the monoculture (Table 1). Leaf ψ_pd_ values for Chinese fir significantly increased with the increase in precipitation. Relatively lower leaf ψ_pd_ values of Chinese fir in mixed forests were observed following different rainfall events (Table 2). As shown in Figure 4, P_n_, G_s_, and T_r_ showed no significant difference for Chinese fir in the pure and mixed forests following light rainfall. In contrast, mixed forests significantly increased P_n_, G_s_, and T_r_ in Chinese fir during moderate and heavy rainfall (*p* < 0.05). In addition, the fine-root biomass (FB) varied in the vertical soil profiles (Table 3). In the shallow soil layers, the FB of Chinese fir exhibited a trend of PC > MCC > MCA. However, at soil depths within 40–100 cm, the FB of Chinese fir in the mixed forests (MCC and MCA) was greater than that observed in the pure forest, with significant differences in the 80–100 cm layers (*p* < 0.05).

**Table 1:** Characteristics of Chinese fir in different plantation types.

Variable	PC	MCC–C	MCA–C
DBH (cm)	23.32 ± 1.01a	24.67 ± 1.72a	23.33 ± 2.43a
Leaf biomass (kg)	100.79 ± 11.51b	191.50 ± 11.52a	179.32 ± 32.21a

Statistically significant differences of each variable among different Chinese fir plantations are denoted by lowercase letters (*p* < 0.05).

**Table 2:** Leaf water potential (ψ_pd_, MPa) for Chinese fir in different plantation types.

Variable	Plantation type	Light rainfall	Moderate rainfall	Heavy rainfall
ψ_pd_	PC	−0.46 ± 0.03Ca	−0.33 ± 0.05Ba	−0.26 ± 0.03Aa
	MCC–C	−0.49 ± 0.03Ca	−0.40 ± 0.05Ba	−0.31 ± 0.02Aa
	MCA–C	−0.51 ± 0.07Ba	−0.41 ± 0.06ABa	−0.37 ± 0.06Aa

Significant differences in leaf *ψ* among precipitation events are indicated by different capital letters in the same row, and significant differences in leaf *ψ* among different Chinese fir plantations are denoted by different lowercase letters within the same column (*p* < 0.05).

**Table 3:** Fine-root biomass and soil water content for the three Chinese fir plantations.

Variable	Layers (cm)	PC	MCC–C	MCA–C
Fine root biomass (g m^−2^)	0–20	100.81 ± 13.77a	74.59 ± 7.39ab	44.92 ± 30.14b
	20–40	83.44 ± 18.45a	73.25 ± 16.42a	58.91 ± 12.56a
	40–60	64.65 ± 8.57a	81.96 ± 20.61a	82.43 ± 28.48a
	60–80	52.75 ± 1.29b	64.09 ± 5.50a	59.81 ± 3.23ab
	80–100	20.17 ± 3.69b	41.22 ± 6.15a	41.68 ± 6.84a
Soil water content (%)	0–20	26.08 ± 3.08b	29.28 ± 4.96a	28.60 ± 5.39a
	20–40	25.86 ± 2.84a	26.90 ± 3.70a	26.92 ± 4.19a
	40–60	23.64 ± 4.85b	26.08 ± 3.81a	26.64 ± 3.48a
	60–80	23.65 ± 3.76b	24.72 ± 3.36ab	25.13 ± 2.86a
	80–100	22.96 ± 3.48b	24.45 ± 3.13a	24.92 ± 3.17a

Statistically significant differences of variables among different Chinese fir plantations are expressed in lowercase letters (*p* < 0.05).

As for soil properties, the SWC in the mixed forests was significantly higher than that in the pure forest except for the 20–40 cm depth (*p* < 0.05; Table 3). Compared to the pure forest, the mixed forests presented significantly higher FC and TP, except for the 60–80 cm depth (Supplementary Table S5). Moreover, most of these soil parameters did not differ in the two mixed forests (Supplementary Table S5).

### Relationships of Chinese fir water uptake proportion with plant and soil properties

**Figure 5:**
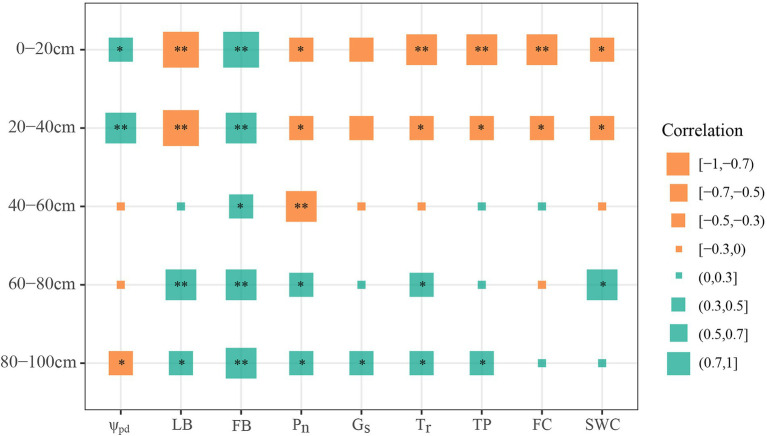
Correlation analysis of the water percentage of Chinese fir absorbed from different soil layers with plant and soil properties. The size of the square represents the magnitude of the values of correlation coefficients. ** and * indicate *p* < 0.01 and *p* < 0.05, respectively. LB, leaf biomass; ψ_pd_, predawn leaf water potential; P_n_, photosynthetic rate; G_s_, stomatal conductance; T_r_, transpiration rate; FB, fine-root biomass; TP, total porosity; SWC, soil water content; FC, field capacity.

We conducted a correlation analysis to examine the relationships between the plant/soil factors and the water uptake percentage of Chinese fir (Figure 5). The percentage of water that Chinese fir extracted from each soil layer was significantly affected by the plant and soil properties. More specifically, the findings showed that the percentage of water absorbed by Chinese fir within the 40 cm soil layer was positively related to ψ_pd_ and FB but was negatively related to LB, P_n_, T_r_, TP, FC, and SWC, respectively (*p* < 0.05). The percentage of water uptake from the 40–60 cm soil layer exhibited a significant correlation solely with FB (*r*^2^ = 0.41, *p* = 0.033) and P_n_ (*r*^2^ = −0.52, *p* = 0.006). In contrast to the shallow soil layers, the percentage of water acquired by Chinese fir from the 60–80 and 80–100 cm soil layers was negatively correlated with ψ_pd_, while it was positively correlated with LB, FB, P_n_, and T_r_. In addition, the percentage of water absorbed by Chinese fir at 60–80 cm within the soil was positively related to SWC (*p* < 0.05). Similarly, the percentage of water absorbed by Chinese fir from the 80–100 cm soil layer was significantly positively related to G_s_ and TP (*p* < 0.05).

### Main factors affecting Chinese fir water uptake patterns

**Figure 6:**
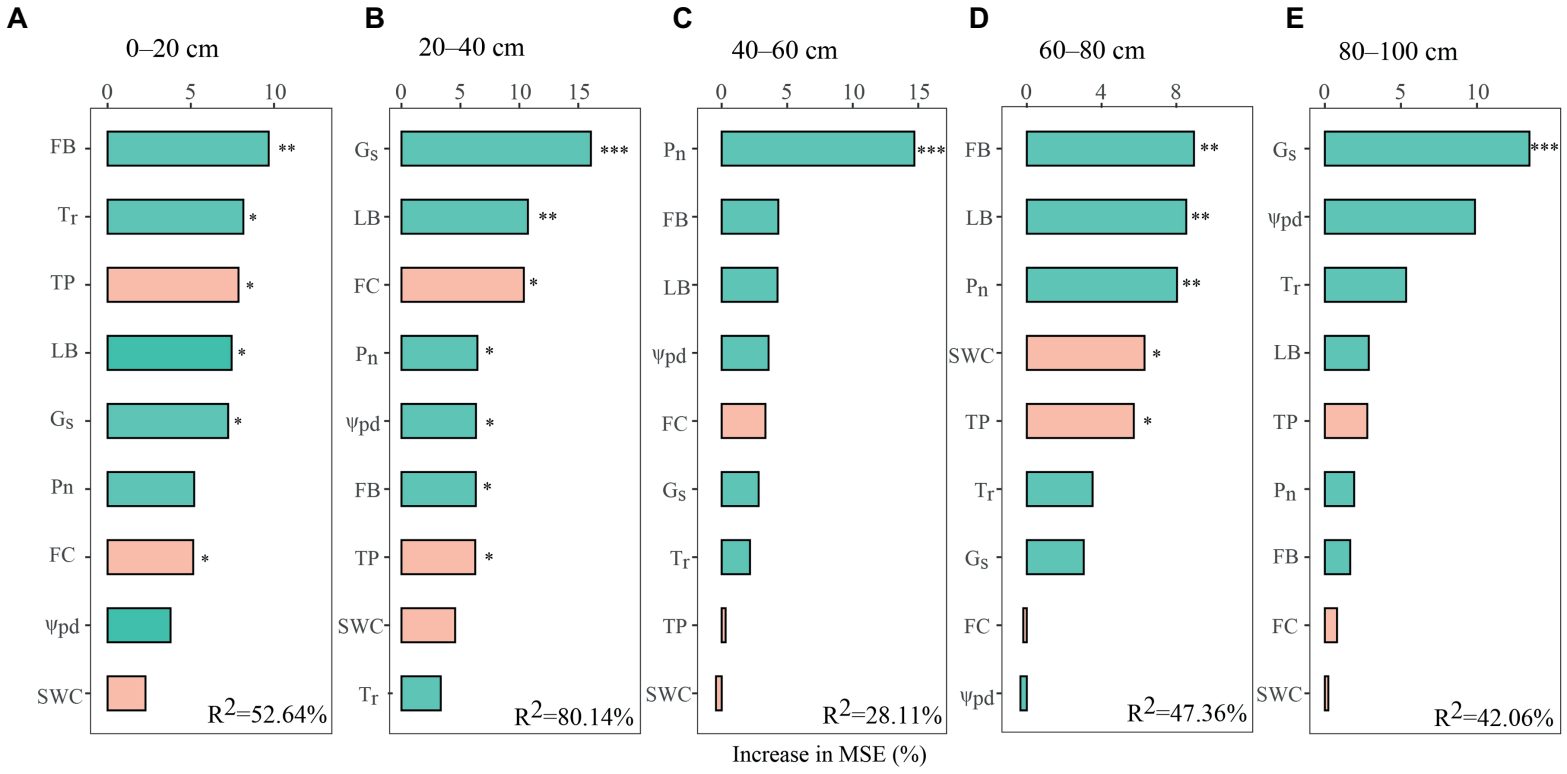
Relative importance of influencing factors to proportion of water absorption based on random forest modeling. The green and orange bars represent plant and soil factors, respectively. The relative importance of the influencing factors is expressed as a percentage of the increased mean squared error (% MSE). Also, ***, **, and * indicate *p* < 0.001, *p* < 0.01, and *p* < 0.05, respectively. LB, leaf biomass; P_n_, photosynthetic rate; G_s_, stomatal conductance; T_r_, transpiration rate; ψ_pd_, predawn leaf water potential; FB, fine-root biomass; TP, total porosity; SWC, soil water content; FC, field capacity.

To discern the potential dominant factors of water-use patterns for Chinese fir, the relative importance of plant and soil properties was evaluated with a random forest analysis (Figure 6). The above biotic and abiotic factors explained 27.06%–79.83% of the variance in the percentage of water acquired by Chinese fir from five soil layers. We found that FB best predicted the proportion of water uptake of Chinese fir for the 0–20 cm soil layers, and G_s_, LB, and FC were best for the 20–40 cm soil layers (Figures 6A,B). Additionally, the main predictor of the percentage of water absorbed by Chinese fir for the 40–60 cm soil layers was P_n_, while FB, LB, G_s_, and ψ_pd_ were the main predictors for deep soil layers (Figures 6C,E). Thus, the tree attributes explained the variance in the water uptake proportions of Chinese fir to a greater extent than the soil properties. Overall, fine-root biomass, leaf biomass, and physiological regulation (i.e., G_s_ and ψ_pd_) were the most prominent factors affecting the water uptake patterns of Chinese fir.

## Discussion

### Water-use patterns of Chinese fir in different plantation types

As expected, the xylem water isotopes of Chinese fir varied significantly between pure and mixed forests in our study (Figure 2). This observation was in accordance with prior studies (Moreno‐Gutiérrez et al., 2012; Wang et al., 2020), suggesting that plant water-use patterns were strongly influenced by forest type. Previous studies in mixed forests revealed that conifers tended to take up shallow soil water, whilst hardwoods relied on relatively stable deeper levels because of their greater competitiveness and ecological adaptability to establish broader and deeper root systems (Castillo et al., 2016; Martín-Gómez et al., 2017). However, the output of the MixSIAR model in our study demonstrated that Chinese fir in the pure forest acquired a greater percentage of water from shallow soil, whereas those in MCC and MCA primarily obtained water from deep soil. This contradiction could be attributed to species differences and species-specific hydrological niche segregation (Brum et al., 2018). Indeed, ecohydrological niche segregation is thought to be an important mechanism for the coexistence of dominant species in mixtures (Silvertown et al., 2015; Wang et al., 2020). Competition among mixed species leads to shallow-rooted species that acquire water from shallow soil and deep-rooted species that shift to extract water from deep soil layers (Grossiord et al., 2017). Therefore, the Chinese fir, an evergreen coniferous deep-rooted species, had easier access to deep soil water when competing for water with other species (i.e., *C. camphora*, *A. cremastogyne*) in the mixture. In contrast, Chinese fir in the pure forest preferentially utilized water from shallow soil, probably because the shallow soil water was more readily accessible and required less energy for absorption (Williams and Ehleringer, 2000). Another important reason might be ascribed to the local precipitation and soil moisture. Specifically, this study area is under the subtropical monsoon climate with a mean annual precipitation of 1,200–1,400 mm. The high precipitation would contribute to a relatively high soil water content, which could favor the development of shallow roots of the pure Chinese fir forest (Table 3), thereby increasing the consumption of shallow soil water. However, even such climatic conditions could not avoid seasonal droughts that occur from time to time (Zhou et al., 2011), and the over-dependence on the shallow soil water of Chinese fir in the pure forest threatened its survival (Bucci et al., 2005; Zhou et al., 2013). In comparison, the mixed Chinese fir forests comprehensively utilized water from different soil layers, which improved water-use efficiency and reduced water loss during precipitation. And the deep roots of Chinese fir in mixed stands could thus ensure the water balance in seasonal drought (Voltas et al., 2015). On the other hand, the shift in water origin from topsoil to deep soil implied the ecological plasticity of plants regarding water use (Valladares et al., 2007), which conferred greater adaptability to environmental changes (Wang et al., 2017). Therefore, Chinese fir in mixed forests exhibits considerable ecological plasticity and is more resistant to changes in subtropical precipitation regimes.

### Drivers of the water-use patterns in Chinese fir

Our results demonstrated that the high plasticity of water-use patterns for Chinese fir was intimately associated with the vertical distribution of roots in each soil layer (Figures 5, 6). This may be attributed to the dimorphic root systems of Chinese fir, permitting them to adjust water-use patterns according to ambient water conditions in seasonally dry subtropical ecosystems (Dawson and Pate, 1996; Liu et al., 2010; Yang et al., 2015). Moreover, traits within the rhizosphere of trees played an essential role in water acquisition for various physiological functions (Moreno‐Gutiérrez et al., 2012; Brum et al., 2018). In this study, fine-root biomass was the main factor influencing the water uptake proportion of Chinese fir in pure and mixed forests from the respective soil layers. This observation could be explained by the following two reasons. First, fine roots with great surface area and strong physiological activity are the primary organ for water and nutrient absorption (McCormack et al., 2015; Hu et al., 2021). Second, the redistribution of fine roots within the soil profile and the maintenance of their metabolic activities are essential for water uptake and the adaptation of plants to variable environments (Schenk, 2008; Kulmatiski and Beard, 2012; Zhang et al., 2020). Hence, the Chinese fir in our mixed forests (MCC and MCA, respectively) had greater fine-root biomass in deep soils and accordingly improved the absorption of deep soil water. These findings further confirmed that Chinese fir is a tree species with a vast ability to exploit water from deep soil, which would promote its survival opportunities and environmental adaptability in plant communities.

In addition to the fine-root biomass, we found that the water-use patterns of Chinese fir were significantly influenced by physiological regulation. Specifically, the proportion of deep soil water uptake by Chinese fir exhibited positive correlations with G_s_ and T_r_, and negative correlations with leaf ψ_pd_. This was consistent with the findings observed in a prior study (O’Keefe et al., 2019), implying that the Chinese fir needed to increase the proportion of deep soil water to meet its transpiration demand through stomatal regulation and leaf ψ decrease. More importantly, tree species could adjust their phenotypic plasticity (i.e., stomatal conductance and leaf water potential, which are generally considered the direct physiological expression of plant water status) to regulate hydraulic traits and water-use patterns (Sperry and Hacke, 2002; Ryel et al., 2004; West et al., 2007). In fact, Chinese fir in the pure forest displayed relatively higher ψ values and lower G_s_ and T_r_, implying that despite a certain percentage of deep soil water being absorbed, it was insufficient to compensate for transpiration losses in the growing season. Thus, Chinese fir in the pure forest limited the transpiration rate by optimizing stomatal control to ensure stable and continuous water supply and physiological metabolism activities, which was supported by its relatively lower leaf carbon concentrations (Supplementary Table S6). Conversely, the lower leaf ψ_pd_ of Chinese fir in mixed forests promoted deep soil water uptake, allowing for high stomatal conductance, thereby improving leaf transpiration and carbon assimilation. These observations reinforce the general knowledge that water from deep soil layers contributes more to the transpiration of Chinese fir during its growing season than water from shallow soil (Yang et al., 2017).

Furthermore, leaf biomass appeared to be a key factor in shaping plant water use. Interestingly, we found that leaf biomass had an opposite effect on the percentage of water absorbed by Chinese fir between shallow and deep soils (Figure 5). This was consistent with the findings of Zhang et al. (2020), who noted that the contrary effect was associated with transpiration. Given that transpiration is the driving force of plant water uptake and that the strength of transpiration is generally reflected by leaf biomass (Forrester et al., 2010; Rothfuss and Javaux, 2017), a smaller leaf biomass of Chinese fir in the pure forest implied a weaker driving force for transpiration and water absorption, thus promoting the utilization of shallow soil water (Table 1; Figure 3). Conversely, higher leaf biomass in taller trees, according to the allometric growth model, tended to have a less negative leaf water potential, implying a narrower leaf-to-soil water potential gradient (Bucci et al., 2005) and a stronger driving force of plant water uptake for transpiration, which could facilitate plant to use of deep soil water (Markewitz et al., 2010). Therefore, the higher leaf biomass of Chinese fir found in mixed forests had a greater percentage of deep soil water uptake than in the pure forest. Additionally, some studies indicated that greater light interception by trees with higher leaf biomass allowed them to afford the carbon costs of deeper fine-root growth (Kim et al., 2016; Brum et al., 2018). These explained why Chinese fir in mixed forests extracted a higher percentage of deep soil water.

Soil properties (including total porosity and field capacity) can mediate tree water-use patterns via regulating its hydraulic conductivity and water holding capacity (Metzger et al., 2017; Zhang et al., 2021). Notably, we found that soil properties were not the main drivers regulating Chinese fir water uptake (Figure 6). This could be ascribed to the fact that soil hydraulic parameters can significantly affect plant water-use patterns only when soil water content is a limiting factor (Coners and Leuschner, 2005). In this study, the SWC of these three stands dropped to thresholds for optimal plant supply (0.5–0.8 times field capacity) during the sampling period (Feddes et al., 2001; Volkmann et al., 2016). Therefore, although mixed Chinese fir forests altered water holding capacity of the soil (Zhang et al., 2022), this was insufficient to significantly influence Chinese fir water uptake patterns. In summary, soil properties exerted smaller impact on Chinese fir water-use patterns than plant properties, which was consistent with findings in other planted forests (Wang et al., 2020; Zhang et al., 2020). Studies have shown that soil properties (i.e., hydraulic parameters) could indirectly modulate plant water-use patterns by modifying and/or collaborating with plant properties (Agee et al., 2021), which might explain the relatively low contribution of soil properties to the water-use patterns of Chinese fir.

### Implications

Our study demonstrated that Chinese fir in the pure forest was more sensitive to precipitation and relied mainly on highly changeable shallow soil water supplied by recent rainwater during the growing season. Although this water-use pattern enabled plants to maximize rainfall use, it could negatively impact the resilience of trees to frequent extreme weather and seasonal drought, especially in the subtropics. In contrast, when grown in mixed forests, Chinese fir shifted their water sources downward and primarily utilized deeper, more stable soil water by changing their properties; therefore, making them less susceptible to water stress. This adaptability promoted forest productivity and carbon sink capacity. The above explanation was supported by our field observations, indicating that the mixed forests had significantly higher above-ground tree biomass compared to the monoculture plantation (Supplementary Table S7). Additionally, our study indicated partial niche complementarity for water sources between Chinese fir and *C. camphora* or *A. cremastogyne* in the mixed forests (Supplementary Table S8; Supplementary Figure S1). This minimizes competition between these dominant co-occurring plants, thereby promoting the stable coexistence of species within the mixture (Silvertown et al., 2015). Therefore, the allocation of mixed species should be prioritized for future plantation construction and management. A rational mixture of different functional tree species improves biodiversity, reduces the impact of seasonal water limitation, and resists climate change impacts by optimizing the efficient use of water and resources, ultimately increasing the productivity and stability of forest ecosystems.

## Conclusion

In this study, the effects of plantation types on water-use patterns of Chinese fir are determined by the MixSIAR model and based on isotopic values of *δ*D and *δ*^18^O. We find that the water-use patterns of Chinese fir vary with the type of plantation. Compared with the monoculture plantation, Chinese fir in mixed plantations acquire more water from deep soil, thereby reducing the utilization of water from shallow soil. The differences in plant water-use patterns among different plantation types are affected by tree attributes. Our findings suggest that Chinese fir in the mixed forests could improve water absorption from deep soil by regulating its properties (i.e., leaf biomass, eco-physiological traits, and fine-root biomass). Thus, the vast capacity of Chinese fir in mixed forests to exploit deep soil water renders them more adaptable to increased extreme precipitation and seasonal drought events in the subtropics, thereby improving the stability and productivity of the community. Moreover, we find that the response of tree water-use patterns in pure and mixed forests varies with precipitation, highlighting the importance of various precipitation levels in shaping tree water-use patterns. Therefore, we recommend that the effects of different magnitudes of precipitation should be studied in the future so that the interactions between tree species and variable hydrologic conditions can be elucidated in detail. These findings enhance our understanding of plant-water relationships, which is necessary for the selection of appropriate tree species for vegetation restoration in this region. This study provides valuable information for water-related vegetation restoration and afforestation management in subtropical areas.

## Data availability statement

The original contributions presented in the study are included in the article/Supplementary material, further inquiries can be directed to the corresponding authors.

## Author contributions

QX and BZ conceived and designed the project. YZ, TW, DG, WX, and RR participated in field sampling and experimental analysis. YZ analyzed the data and drafted the original manuscript. QX, BZ, TW, and SW helped revise the manuscript. All authors contributed to the article and approved the submitted version.

## Funding

This study was supported by the National Natural Science Foundation of China (31870716 and 31670720) and the National Nonprofit Institute Research Grant of CAF (CAFYBB2017ZB003 and CAFYBB2021ZE002).

## Conflict of interest

The remaining authors declare that the research was conducted in the absence of any commercial or financial relationships that could be construed as a potential conflict of interest.

The reviewer XW declared a shared affiliation with the authors YZ, QX, BZ, DG, TW, WX, and RR to the handling editor at the time of review.

## Publisher’s note

All claims expressed in this article are solely those of the authors and do not necessarily represent those of their affiliated organizations, or those of the publisher, the editors and the reviewers. Any product that may be evaluated in this article, or claim that may be made by its manufacturer, is not guaranteed or endorsed by the publisher.
