# Supplementary material for: Contrasting water-use patterns of Chinese fir among different plantation types in a subtropical region of China
Source: Front Plant Sci. 2022 Sep 15;13:946508. doi: 10.3389/fpls.2022.946508 (PMC9520624; doi:10.3389/fpls.2022.946508)
Supplement: Supplementary file 1 [file Data_Sheet_1.docx]

Supplementary Material

Supplementary Information

Contents of this file:

Table S1 to S8

Figure S1

## Supplementary Tables

**Table S1** Soil texture for the study site.

| Layers (cm) | Sand (%) | Silt (%) | Clay (%) |
| --- | --- | --- | --- |
| 0-20 | 8.00 ± 2.69 | 51.34 ± 2.88 | 40.65 ± 2.90 |
| 20-40 | 1.48 ± 0.50 | 52.33 ± 3.49 | 46.17 ± 3.44 |
| 40-60 | 3.08 ± 2.13 | 46.86 ± 4.97 | 50.04 ± 3.11 |
| 60-80 | 2.19 ± 0.97 | 45.49 ± 4.16 | 52.30 ± 3.18 |
| 80-100 | 1.46 ± 0.91 | 44.40 ± 4.65 | 54.13 ± 3.86 |

**Table S2** The soil fertility parameters in the three Chinese fir plantations.

| Variables | Layers (cm) | PC | MCC | MCA |
| --- | --- | --- | --- | --- |
| Total N  (mg/g) | 0-20 | 1.35±0.30a | 1.38±0.07a | 1.44±0.17a |
|  | 20-40 | 1.10±0.17a | 1.13±0.24a | 0.98±0.21a |
|  | 40-60 | 1.01±0.18a | 0.84±0.10a | 0.86±0.17a |
|  | 60-80 | 0.81±0.10a | 0.84±0.09a | 0.86±0.14a |
|  | 80-100 | 0.90±0.18a | 0.79±0.05a | 0.85±0.20a |
| Total P  (mg/kg) | 0-20 | 284.57±19.75a | 259.35±34.92a | 233.11±17.02a |
|  | 20-40 | 261.47±29.30a | 230.92±34.72a | 211.99±14.17a |
|  | 40-60 | 232.10±26.85a | 203.19±20.44a | 211.57±25.76a |
|  | 60-80 | 256.47±21.56a | 211.70±24.30a | 233.08±37.02a |
|  | 80-100 | 229.74±28.64a | 222.11±1.68a | 202.29±21.78a |
| Total K  (mg/kg) | 0-20 | 2366.18±219.29a | 2084.65±79.56a | 2334.05±250.52a |
|  | 20-40 | 2586.33±261.44a | 2152.93±49.41a | 2329.22±312.96a |
|  | 40-60 | 2596.02±192.51a | 2312.40±158.85a | 2518.00±113.29a |
|  | 60-80 | 2932.17±199.69a | 2514.84±222.34a | 2589.09±181.21a |
|  | 80-100 | 2771.12±76.27a | 2771.12±260.30a | 2662.60±132.38a |

The results are reported as means ± SD. The different lowercase letters in the same row indicate significant differences at a 0.05 probability level. PC: pure *C. lanceolata* plantation; MCC: mixed plantation with *C. lanceolata* and *C. camphora*; MCA: mixed plantation with *C. lanceolata* and *A. cremastogyne*.

**Table S3** Summary of meteorological parameters for the measurement dates of tree and soil factors.

| Indicators | Date | Temperature (°C) | Relative humidity (%) | Barometric pressure (hPa) |
| --- | --- | --- | --- | --- |
| Tree | 15/8/2020 | 26.45 | 79 | 946.0 |
|  | 20/9/2019 | 21.91 | 75 | 955.5 |
|  | 03/10/2019 | 26.40 | 56 | 951.0 |
| Soil | 23/7/2019 | 27.65 | 79 | 943.1 |

**Table S4** Comparison of isotopic composition in xylem water of Chinese fir in different plantation types after three rainfall events.

| Rainfall events | Isotopic composition | df | F–value | P–value |
| --- | --- | --- | --- | --- |
| Light rainfall | *δ*^2^H | 2 | 2.323 | 0.107 |
|  | δ^18^O | 2 | 2.612 | 0.082 |
| Moderate rainfall | *δ*^2^H | 2 | 4.220 | 0.023 |
|  | δ^18^O | 2 | 8.048 | 0.001 |
| Heavy rainfall | *δ*^2^H | 2 | 3.931 | 0.026 |
|  | δ^18^O | 2 | 17.525 | 0.000 |

**Table S5** The soil properties among the three plantations.

| Variable | Layers (cm) | PC | MCC | MCA |
| --- | --- | --- | --- | --- |
| Field capacity  (%) | 0–20 | 35.01 ± 0.99 b | 41.48 ± 1.19 a | 40.69 ± 3.61 a |
|  | 20–40 | 28.75 ± 1.34 c | 38.23 ± 0.56 a | 35.10 ± 1.37 b |
|  | 40–60 | 27.88 ± 1.91 b | 42.33 ± 3.65 a | 31.99 ± 1.57 a |
|  | 60–80 | 34.49 ± 4.10 a | 35.98 ± 4.61 a | 31.58 ± 1.44 a |
|  | 80–100 | 25.27 ± 3.50 b | 32.18 ± 0.48 a | 36.82 ± 3.18 a |
| Total porosity  (%) | 0–20 | 45.79 ± 0.60 b | 50.90 ± 2.45 a | 49.58 ± 1.49 a |
|  | 20–40 | 39.07 ± 1.82 b | 48.03 ± 3.08 a | 45.34 ± 2.96 a |
|  | 40–60 | 39.68 ± 2.50 a | 46.65 ± 5.81 a | 43.31 ± 1.56 a |
|  | 60–80 | 43.70 ± 1.00 a | 46.65 ± 3.41 a | 44.62 ± 0.27 a |
|  | 80–100 | 40.43 ± 2.44 b | 46.82 ± 1.02 a | 45.24 ± 1.67 a |

The results are reported as means ± SD. The different lowercase letters in the same row indicate significant differences at a 0.05 probability level. PC: pure *C. lanceolata* plantation; MCC: mixed plantation with *C. lanceolata* and *C. camphora*; MCA: mixed plantation with *C. lanceolata* and *A. cremastogyne*.

**Table S6** Leaf organic carbon concentration (g/kg) of Chinese fir among the three plantations.

| Plantation type | Light rainfall | Moderate rainfall | Heavy rainfall |
| --- | --- | --- | --- |
| PC | 47.77±0.45a | 46.00±0.30b | 46.36±0.28b |
| MCC–C | 47.95±0.31a | 46.86±0.58a | 47.83±1.15a |
| MCA–C | 47.98±0.45a | 46.13±0.52a | 48.46±0.72a |

The results are reported as means ± SD. The different lowercase letters in the same row indicate significant differences at a 0.05 probability level. PC: *C. lanceolata* in the pure plantation; MCC–C: *C. lanceolata* in the mixed plantation with *C. lanceolata* and *C. camphora* and MCA–C: the *C. lanceolata* in the mixed plantation with *C. lanceolata* and *A. cremastogyne*.

**Table S7** Tree biomass among the three Chinese fir plantations.

|  | PC | MCC | MCA |
| --- | --- | --- | --- |
| Tree biomass (kg·m^−2^) | 10.34 ± 1.69 b | 20.14 ± 0.93 a | 22.46 ± 1.00 a |

The results are reported as means ± SD. The different lowercase letters in the same row indicate significant differences at a 0.05 probability level. PC: pure *C. lanceolata* plantation; MCC: mixed plantation with *C. lanceolata* and *C. camphora*; MCA: mixed plantation with *C. lanceolata* and *A. cremastogyne*.

**Table S8** Comparison of isotopic compositions in xylem water for mixed stands during sample period.

| Rainfall events | Plantations | Isotopic composition | df | F–value | P–value |
| --- | --- | --- | --- | --- | --- |
| Light  rainfall | MCC | H | 34 | 0.966 | 0.005 |
|  |  | O | 34 | 2.141 | 0.000 |
|  | MCA | H | 34 | 3.295 | 0.022 |
|  |  | O | 34 | 4.479 | 0.004 |
| Moderate rainfall | MCC | H | 22 | 16.296 | 0.001 |
|  |  | O | 22 | 1.480 | 0.020 |
|  | MCA | H | 22 | 0.007 | 0.000 |
|  |  | O | 22 | 0.138 | 0.050 |
| Heavy  rainfall | MCC | H | 34 | 1.181 | 0.000 |
|  |  | O | 34 | 1.319 | 0.000 |
|  | MCA | H | 34 | 0.982 | 0.001 |
|  |  | O | 34 | 2.020 | 0.037 |

Tree xylems include Chinese fir, *C. camphora* and *A. cremastogyne*. PC: pure *C. lanceolata* plantation; MCC: mixed plantation with *C. lanceolata* and *C. camphora*; MCA: mixed plantation with *C. lanceolata* and *A. cremastogyne*.


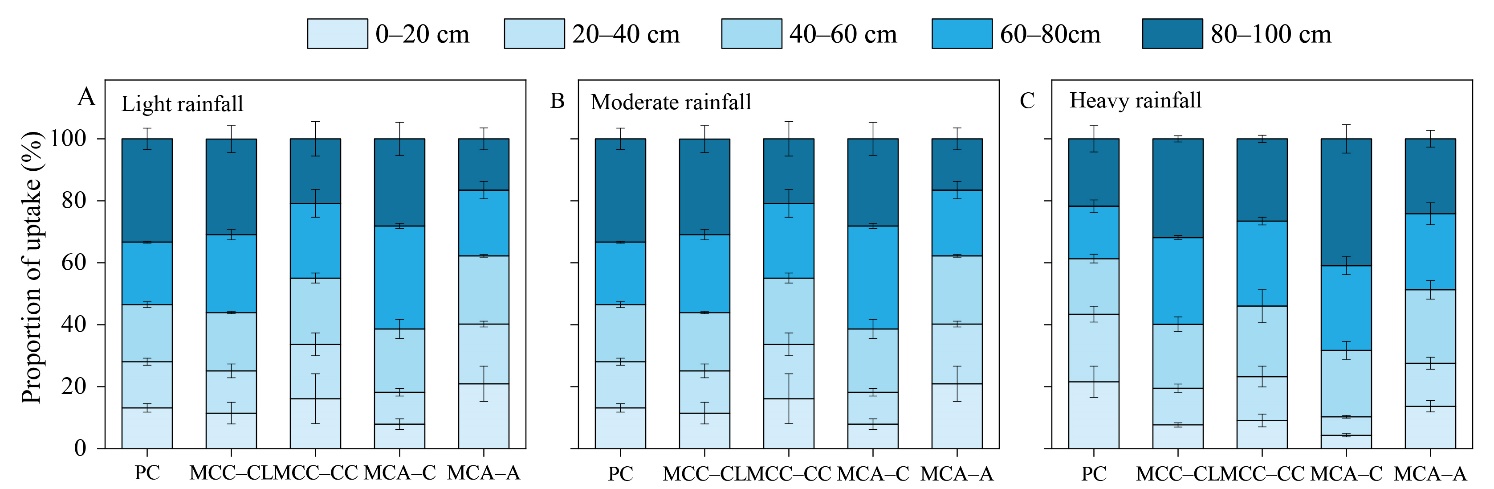


**Figure S1** Variations in proportion of water uptake by each species in the three plantations following (A) light, (B) moderate and (C) heavy rainfall events. Error bars are expressed as the standard deviation. PC: pure *C. lanceolata* plantation; MCC-CL: *C. lanceolata* in the mixed plantation with *C. lanceolata* and *C. camphora*; MCC-CC: *C. camphora* in the mixed plantation with *C. lanceolata* and *C. camphora*; MCA-C: *C. lanceolata in* the mixed plantation with *C. lanceolata* and *A. cremastogyne*; MCA-A: *A. cremastogyne* in the mixed plantation with *C. lanceolata a*nd *A. cremastogyne*.
